# Cowpea and Groundnut Haulms Fodder Trading and Its Lessons for Multidimensional Cowpea Improvement for Mixed Crop Livestock Systems in West Africa

**DOI:** 10.3389/fpls.2017.00030

**Published:** 2017-01-31

**Authors:** Anandan Samireddypalle, Ousmane Boukar, Elaine Grings, Christian A. Fatokun, Prasad Kodukula, Ravi Devulapalli, Iheanacho Okike, Michael Blümmel

**Affiliations:** ^1^International Livestock Research InstituteIbadan, Nigeria; ^2^International Institute of Tropical AgricultureKano, Nigeria; ^3^Office of Agriculture Research and Policy, Bureau for Food Security, United States Agency for International DevelopmentWashington, DC, USA; ^4^International Institute of Tropical AgricultureIbadan, Nigeria; ^5^International Livestock Research InstitutePatancheru, India; ^6^International Livestock Research InstituteAddis Ababa, Ethiopia

**Keywords:** dual purpose cowpea, haulm fodder quality, multi-dimensional crop improvement, heritability of haulm traits, cowpea fodder, groundnut fodder

## Abstract

Cowpea is an important legume crop in Africa, valued highly for its grain and also haulms, which are a tradable commodity in fodder markets. Fodder market surveys in Northern Nigeria showed that groundnut haulms were priced higher than cowpea haulms, probably because of their superior nutritive value. The economic value of haulms has prompted cowpea breeders and livestock nutritionists to explore haulm fodder traits as additional selection and breeding criteria. Fifty cowpea genotypes cultivated across five locations in Nigeria in 2013 and 2014 were evaluated for food fodder traits. Significant (*P* < 0.05) genotypic dependent variations were observed in yields (kg/ha) of grains (537–1082) and haulms (1173–3368), though significant (*P* < 0.05) effects of location and year were observed. Grain and fodder yield had a tendency to be positively correlated (*r* = 0.26, *P* = 0.07). Haulms were analyzed for nitrogen (N), fiber fractions, *in vitro* digestibility, and metabolizable energy content. Highly significant variations were observed in all genotypic and livestock nutrition traits, although location and year had significant effects. Trade-offs between grain yield and haulm fodder quality traits were largely absent and haulm acid detergent lignin and grain yield were even inversely correlated (*r* = -0.28, *P* = 0.05), that is high grain yielders had decreased haulm lignin. However, haulm N and grain yield also tended to be negatively associated (*r* = -0.26, *P* = 0.07). Haulm fodder quality traits and haulm yield were mostly positively correlated (*P* < 0.05). Broad sense heritabilities for grain and fodder yield were 0.50 and 0.29, respectively, while heritability for haulm fodder quality traits ranged from 0.61 to 0.67, providing opportunities for concomitant increase in grain yield and haulm fodder quality traits. Selection of the 10 highest ranking genotypes for grain yield, haulm yield, haulm N, and haulm *in vitro* organic matter digestibility showed selection groups overlapping, suggesting that multi-trait selection is feasible. Economical evaluation showed that choice of primary traits is context specific, highlighting the need for identifying and targeting appropriate genotypes to fit different production systems. Considering haulm quantity and quality as traits of economic value can increase overall plant value in mixed crop-livestock systems.

## Introduction

Legumes are important crops of mixed crop-livestock systems, providing highly nutritious food in their grains and highly palatable fodder in their haulms while at the same time contributing to soil fertility through nitrogen fixation (Alemayehu, 1997). Among the legumes, cowpea is a major crop in Africa which accounts for over 95% of the global production, with West Africa being the major regional producer. Promoting the use of cowpea as a dual-purpose crop, providing both grain and fodder, is attractive in mixed crop-livestock systems where land, water and fodder are becoming increasingly scarce and where fodder shortage can become severe, especially in the dry season (Tarawali et al., 1997). While cowpea area and production in West Africa over the last 5 years (2010–2014) have shown steady annual growth rates of 2.32 and 0.74%, respectively, yield per ha has declined at a rate of 1.53% (FAOSTAT, 2014). Thus, while Nigeria, Niger and Burkina Faso are ranked as the first, second and third largest producers of cowpeas globally, the yield per ha in the West Africa region is less than 20% of the average yield of the five top most producing countries (FAOSTAT, 2014). Forecasts by Abate et al. (2012) for the period of 2007–2030 predict that in West and Central Africa the supply rate of cowpea (2.6%) will continue to grow more slowly than the demand rate (2.7%). Thus, the need and scope for improving the productivity of cowpea in West Africa through breeding and management is urgent, with substantial potential impacts on food and fodder security and, consequently, livelihoods in the region. Projections suggest that by 2050 more than 80% of the population of West Africa will live in rain-fed mixed crop-livestock systems (Thornton et al., 2002), where cowpea is an important crop. Cowpea haulms are already an important fodder source for livestock in crop-livestock systems in the Sahel regions of West Africa where feed scarcity and seasonality is the major constraint to improved livestock production (Agyemang, 2002). The increasing demand for livestock product will further increase feed and fodder demand and the shrinking arable land and water resources will raise the importance of by-products like legume haulms as fodder sources even more (Blümmel et al., 2012a). *Ex ante* assessments of the impact of cowpea varieties concomitantly improved for grain and haulm yield and haulm fodder quality suggested multiple benefits and high likely adoption rate of such varieties (Kristjanson et al., 2005). A wide body of work exists showing that concomitant improvement for grain and straw yield and straw quality is feasible in cereals such as barley, maize, rice, sorghum, and pearl millet (Sharma et al., 2010; Blümmel et al., 2012a; Ertiro et al., 2013a,b). Similarly, for groundnut, Nigam and Blümmel (2010), Prasad et al. (2010) and Blümmel et al. (2012b) reported the potential for concomitant improvement of grain yield, haulm yield and haulm fodder quality. Less information is available in cowpea. The present work therefore explores: (1) lessons from legume haulm fodder trading; (2) genotypic variations in grain and haulm yield and haulm fodder quality traits in 50 genotypes of cowpea; (3) the stability of traits across years and locations; (4) trait relationships and tradeoffs; and (5) opportunities of multi-trait improvement of cowpea.

## Materials and Methods

### Fodder Markets Survey

A survey of five fodder markets across Kano state in Nigeria was carried out over 2 years in 2009 and 2010 to understand the seasonality, price structure and price quality relationships in cowpea and groundnut haulms, legumes widely cultivated and traded in Northern Nigeria. The surveys were carried out monthly by visiting fodder markets in Ladi, Gujungu, Garki, Gezawa, and Badume. At the markets, haulm bundles of different sizes varied in price and similar size bundles in same period and market were priced differently due to quality differences perceived by their visual appearance. To account for the differences in quality, the survey teams graded the bundles into categories based on the leaf and stem proportion and color and recorded their prices. A total of 200 observations of the price of haulms and 163 observations of the price of grains were collected during the survey. Haulm bundles were weighed and the samples were collected, dried, ground through 1 mm sieve and analyzed for fodder quality traits using near infrared spectroscopy (NIRS).

### Multi-Location Trials

Fifty cowpea breeding lines selected from IITA nurseries were tested for food and fodder traits. In 2013 and 2014 the lines were grown at five locations representing four agro ecologies: (i) Sahel (Malamadori, Jigawa State in Nigeria and Magaria and Maradi in Niger Republic), (ii) the Sudan Savanna (Minjibir, Kano State in Nigeria), (iii) the Northern Guinea Savanna (Shika, Kaduna State in Nigeria), and (iv) Forest Savannah Transition (Ibadan, Oyo State, in Nigeria). In both years, the experiments were implemented at Malamadori, Minjibir, Shika, and Ibadan. Magaria was added in 2013 while Maradi was added in 2014. A randomized complete block design with three replications was used. The experimental plots consisted of four rows of 4 m length with spacing of 0.75 m between rows and 0.20 m within rows. 100kg/ha of NPK were applied before planting or immediately after planting. Two to three hand weeding and three to four insecticide sprays was applied as soon as weed and insect infestations reached recommended intervention levels. The list of tested genotypes is as follows -Danila, IT00K-1148, IT00K-1263, IT00K-835-45, IT00K-898-5, IT00K-901-5, IT03K-316-1, IT03K-324-9, IT03K-351-1, IT03K-378-4, IT04K-227-4, IT06K-147-1, IT06K-270,IT06K-275, IT06K-335-9, IT07K-279-13, IT07K-291-69, IT07K-318-2, IT90K-277-2,IT95K-1072-57, IT96D-610, IT97K-1042-3, IT97K-1069-6, IT97K-1101-5, IT97K-131-1,IT97K-390-2, IT97K-499-35, IT97K-568-18, IT98D-1399, IT98K-1092-2, IT98K-1103-13,IT98K-1263, IT98K-128-3, IT98K-131-2, IT98K-166-4, IT98K-205-8, IT98K-412-13,IT98K-491-4, IT98K-503-1, IT98K-506-1, IT98K-589-2, IT98K-628, IT99K-216-44, IT99K-377-1, IT99K-494-6, IT99K-529-1, IT99K-529-2, IT99K-573-1-1, IT99K-573-2-1, and IT99K-7-22-2-2.

### Fodder Sample Analysis

Fodder samples from the test lines were collected in paper bags at grain maturity and oven dried in Ibadan or sundried in the other locations. Dried haulms were ground to pass through a 1 mm mesh. All cowpea haulm samples were analyzed for nitrogen (N), neutral (NDF) and acid (ADF) detergent fiber, acid detergent lignin (ADL), *in vitro* organic matter digestibility (IVOMD) and metabolizable energy (ME) content by NIRS, using a NIRS equation specifically developed for cowpea haulms. Nitrogen represents the protein content (crude protein = N × 6.25), while IVOMD represents the potential digestibility of the fodder and is negatively affected by the structural carbohydrates components (NDF, ADF, and ADL). The ME content of fodder estimates the energy available to the animal after accounting for fecal, urinary and methane losses. Nitrogen, IVOMD and ME are positive fodder nutritional quality traits while NDF, ADF, and ADL are negative ones.

### Statistical Analysis

The SAS 9.4 (SAS, 2012) statistical package was used for analysis of variance (ANOVA) by general linear model (PROC GLM). The model for the randomized complete block design was Y_ijk_ = μ+G_i_+E_j_+T_k_+GE_ij_+TE_jk_+GT_ik_+e_ijk_, where μ is the mean, G_i_ the effect of ith genotype, E_j_ the effect of jth environment, T_k_ is effect of kth year, GE_ij_ is the interaction of ith genotype with jth environment, TE_jk_ the interaction of the kth year with jth environment, GT_ik_ the interaction of ith genotype with kth year and e_ijk_ the random error. Comparison of means between treatments was determined using Fisher’s least significance difference (LSD) test at 5% level of significance. Simple correlations among laboratory traits were calculated by PROC CORR. Estimation of heritability and the variance components of the traits were determined using mixed model with restricted maximum likelihood (REML) and PROC VARCOMP.

## Results

### Legume Haulms for Livestock Fodder as Tradable Commodity-Fodder Markets Surveys

Cowpea haulms were sold in bundles of small size (3.8–5.5 kg), while groundnut haulms were sold in bundles of three sizes: small (5–6 kg), medium (11.5–12.5 kg), and large (16–18 kg). Groundnut haulms were available throughout the year in contrast to cowpea haulms, which were available only between January and August. The price of cowpea haulms in small bundles varied between 110 and 160 Naira (0.95–1.06 USD), with an average of 135 Naira (0.89 USD), whereas prices for small bundles of groundnut haulms varied from 400 to 1400 Naira (2.65–9.27 USD) with an average of 620 Naira (4.11 USD). The prices for medium and large bundles of groundnut haulm ranged between 1200 and 2000 Naira (7.95–13.25 USD) with an average of 1678 Naira (11.11 USD) and between 1500 and 3200 Naira (9.93–21.19 USD) with an average of 2440 Naira (16.16 USD), respectively. Small bundles of groundnut haulms were more often available in the market than medium and large ones. Within haulms of the same crop and bundle size, price differed due to perceived visual quality differences. After discussions with the fodder market actors, the survey team graded the haulms into six categories with green and leafy as the best category followed by very good, good, green and stemmy, sun damaged, and rain damaged.

Mean price of cowpea haulms per kg was lower than of groundnut haulms and the price ratio of grain to haulm was much higher in cowpea (3.5 versus 1.1) than in groundnut (**Table 1**). Laboratory fodder quality traits of cowpea haulms were inferior to those of groundnut haulms. Haulm N content was significantly (*P* < 0.01) lower (2.0 versus 2.4%), and NDF content was significantly (*P* < 0.01) higher (59.9 versus 49.3 %) in cowpea compared to groundnut haulms (**Table 2**). Similarly, ADF and ADL tended to be higher and IVOMD and ME tended to be lower in cowpea haulms compared to groundnut haulms.

**Table 1:** Mean monthly haulm prices and price ratio of grains to haulms in cowpea and groundnut surveyed over 2 years from five fodder markets in Northern Nigeria.

	Jan	Feb	Mar	Apr	May	Jun	Jul	Aug	Sep	Oct	Nov	Dec	Average
**Haulms price range (Naira/small bundle)**													
Cowpea	110–140	130–160	120–160	130–140	120–130	120–130	130–150	130–160					
Groundnut	150–1000	400–1200	550–1200	500–1400	500–600	600–1200	500–1000	500–600	400–600	400–600	400–600	400–600	
**Grains price (Naira/kg)**													
Cowpea	105	99	83	105	104	103	101	104	95	79	86	96	97
Groundnut	123	125	109	129	130	131	126	125	98	98	94	114	117
**Haulms price (Naira/kg)**													
Cowpea	27	32	30	29	27	26	29	32					29
Groundnut	106	117	137	143	100	125	115	105	96	92	86	86	109
**Price ratios (Grain:haulms)**													
Cowpea	3.9	3.1	2.7	3.6	3.9	4.0	3.5	3.3					3.5
Groundnut	1.2	1.1	0.8	0.9	1.3	1.0	1.1	1.2	1.0	1.1	1.1	1.3	1.1

*1USD = 151 Naira*.

**Table 2:** Mean haulm fodder quality traits nitrogen (N), neutral (NDF) and acid (ADF) detergent fiber, acid detergent lignin (ADL), *in vitro* organic matter digestibility (IVOMD) and metabolizable energy (ME) content of cowpea haulm (CP) and groundnut haulms (GN) collected monthly over 2 years from five fodder markets in Northern Nigeria.

Haulms	N	NDF	ADF	ADL	IVOMD	ME
CH	2.0^a^	59.9^a^	41.0	8.6	55.6	7.8
GN	2.4^b^	49.3^b^	36.6	7.5	57.1	7.9

*Means with different superscript differ significantly (*P* < 0.05)*.

### Genotype Differences in Grain and Fodder Yield of Cowpea and the Effect of Environment

Mean grain yields of cowpea across genotypes varied 2.01-fold, while the variations across locations and years were 2.09 and 1.09, respectively. Among the locations, Ibadan representing the forest transition zone recorded the highest yields followed by Minjibir (Sudan savanna region), Shika (Northern Guinea savanna region), Malamadori and Magaria, with the latter both located in the Sahel region (**Table 3**). Highly significant (*P* < 0.0001) genotypic differences were observed in grain yield but location and year had highly significant (*P* < 0.01 to *P* < 0.0001) effects (**Table 4**). Interactions between genotype, location, and year for grain yield and yields of N, IVOMD and ME were also significant, with most consistent interactions observed for location × year (**Table 4**).

**Table 3:** Variation in yield of grains (GY) and haulms (HY) and in haulm fodder quality traits nitrogen (N), neutral (NDF) and acid (ADF) detergent fiber, ADL, IVOMD and ME content in 50 genotypes of cowpea grown at five locations and over 2 years.

	Location wise					Year wise		Pooled
Variable	Magaria	Malam-M	Minjibir	Ibadan	Shika	1st year	2nd year	Mean
GY (kg/ha)	512^d^	785^c^	969^b^	1070^a^	964^b^	761^b^	832^a^	797 (537-1082)
HY (kg/ha)	609^d^	1937^b^	2046^b^	1385^c^	2348^a^	1569^b^	2053^a^	1786 (1173-3368)
N (%)	3.0^a^	2.6^b^	2.0^c^	1.8^d^	1.8^d^	2.2^b^	2.3^a^	2.2 (1.5-2.5)
NDF (%)	53.7^e^	67.3^c^	60.4^d^	69.9^a^	68.7^b^	65.8^a^	62.1^b^	64.0 (59.8-71.5)
ADF (%)	28.9^e^	39.7^c^	37.2^d^	49.4^a^	43.0^b^	39.1^b^	40.1^a^	39.6 (36.4-47.3)
ADL (%)	5.9^d^	7.5^c^	7.5^c^	10.2^a^	7.8^b^	7.5^b^	8.0^a^	7.8 (6.8-9.4)
ME (MJ/kg)	8.70^c^	8.89^a^	8.77^b^	7.97^e^	8.48^d^	8.81^a^	8.32^b^	8.57 (8.21-8.82)
IVOMD (%)	61.5^a^	60.2^c^	60.8^b^	55.3^e^	57.8^d^	59.5^a^	58.7^b^	59.1 (56.6-61.0)

*Means with different superscript differ significantly (*P* < 0.05)*.

**Table 4:** Analysis of variance for effects of genotypes (G), location (L) and year (Y) and their interactions for grain (GY) and haulm yield (HY), haulm nitrogen (N) neutral (NDF) and acid detergent fiber (ADF), ADL, IVOMD and metabolisable energy (ME) in 50 cowpea lines grown over 2 years at five locations.

Variable	G	L	Y	G × L	G × Y	L × Y	G × L × Y	h^2^
N	^∗∗∗∗^	^∗∗∗∗^	^∗∗∗∗^	^∗∗∗∗^	^∗∗^	^∗∗∗∗^	^∗∗∗^	0.67
NDF	^∗∗∗∗^	^∗∗∗∗^	^∗∗∗∗^	^∗∗∗∗^	NS	^∗∗∗∗^	^∗∗∗∗^	0.59
ADF	^∗∗∗∗^	^∗∗∗∗^	^∗∗∗∗^	^∗∗∗∗^	^∗∗∗^	^∗∗∗∗^	^∗∗∗∗^	0.69
ADL	^∗∗∗∗^	^∗∗∗∗^	^∗∗∗∗^	^∗∗∗∗^	^∗∗∗∗^	^∗∗∗∗^	^∗∗∗∗^	0.69
ME	^∗∗∗∗^	^∗∗∗∗^	^∗∗∗∗^	^∗∗∗∗^	^∗∗^	^∗∗∗∗^	^∗∗^	0.67
IVOMD	^∗∗∗∗^	^∗∗∗∗^	^∗∗∗∗^	^∗∗∗∗^	^∗∗∗^	^∗∗∗∗^	^∗∗^	0.61
GY	^∗∗∗∗^	^∗∗∗∗^	^∗∗∗∗^	^∗∗∗^	^∗^	^∗∗∗∗^	^∗∗∗^	0.50
HY	^∗∗∗∗^	^∗∗∗∗^	^∗∗^	^∗∗∗^	^∗∗^	^∗∗∗∗^	^∗^	0.29

*Estimates of broad sense heritabilities (h^*2*^) are included. Data are probability levels for the ratio of effect mean square to the appropriate error mean square (^∗^*P* < 0.05, ^∗∗^*P* < 0.01, ^∗∗∗^*P* < 0.001, ^∗∗∗∗^*P* < 0.0001; NS: *P* > 0.05)*.

Greatest haulm yields were observed in Shika followed by Minjibir, Malamadori, Ibadan, and Magaria (**Table 3**). Highly significant (*P* < 0.0001) genotypic differences were observed for haulm yield but location and year had highly significant (*P* < 0.01 to *P* < 0.0001) effects (**Table 4**). Interactions between genotype, location and years were also significant, with most consistent interactions observed for location × year (**Table 4**). The broad sense heritability estimate for grain yield was moderate (*h*^2^ = 0.50) and low (*h*^2^ = 0.29) for haulm yield (**Table 4**).

### Genotype Differences in Haulm Fodder Quality Traits of Cowpea and the Effect of Environment

Some haulm fodder quality traits have positive relations to livestock nutrition, such as N, ME, and IVOMD, which should be high or negative, such as for the fiber fractions NDF, ADF, and ADL, which should be low. Across locations and years differences in fodder quality traits observed between genotypes were greatest for N followed by fiber constituents and finally IVOMD and ME (**Table 3**). Highly significant (*P* < 0.0001) variations were observed for all fodder quality traits (**Table 4**). Except for one case, interactions between genotype, location and years were also significant, with most consistent interactions observed for location × year (**Table 4**) effects. Broad sense heritabilities for haulm fodder quality traits ranged from 0.59 to 0.69. Broad sense heritabilities for yields of N, IVOMD and ME ranged from 0.31 to 0.39 (**Table 4**).

### Relationships between Grain and Haulm Traits

Across locations and years, grain yield and fodder yield tended to be (*P* = 0.07) positively correlated (**Table 5**). Haulm fodder quality traits and grain yields were largely unrelated, except for ADL which was significantly (*P* < 0.05) negatively correlated with grain yield. Correlation of fodder yield and quality attributes revealed that N and NDF were not significant. Haulm yields were significantly (*P* < 0.01) negatively correlated with ADF and ADL and positively correlated with IVOMD (*P* < 0.01) and ME (*P* < 0.05).

**Table 5:** Inter correlations between grain yields (GY), haulm yields (HY), nitrogen (N) neutral (NDF) and ADF, ADL, IVOMD and metabolisable energy (ME) in 50 cowpea lines grown over 2 years at five locations.

	N	NDF	ADF	ADL	ME	IVOMD	GY	HY
N	1.00	-0.83 (0.0001)	-0.68 (0.0001)	-0.46 (0.001)	0.13 (0.37)	0.52 (0.0001)	-0.26 (0.07)	0.21 (0.14)
NDF		1.00	0.78 (0.0001)	0.70 (0.0001)	-0.21 (0.14)	-0.56 (0.0001)	0.04 (0.77)	-0.22 (0.12)
ADF			1.00	0.90 (0.0001)	-0.69 (0.0001)	-0.90 (0.0001)	-0.10 (0.51)	-0.45 (0.001)
ADL				1.00	-0.66 (0.0001)	-0.82 (0.0001)	-0.28 (0.05)	-0.56 (0.0001)
ME					1.00	0.90 (0.0001)	0.23 (0.11)	0.35 (0.01)
IVOMD						1.00	0.11 (0.44)	0.44 (0.001)
GY							1.00	0.26 (0.07)
HY								1.00

## Discussion

### Findings from Fodder Market Surveys Relevant for Crop Improvement

Surveys of crop residues sold in fodder markets can yield important information for crop improvement (Blümmel and Rao, 2006; Sharma et al., 2010; Teufel et al., 2010). The fact that crop residues are a tradable commodity and constitute a value chain requiring collection from the field, transport by middlemen and trading by wholesaler and retailers, is remarkable and challenges the notion of crop residues as minor by-products or even waste products. The cost of stover, straws, and haulms relative to the primary product of grains can inform crop improvement about whole plant value. Demand for fodder is high during the dry season and farmers in Nigeria may earn up to 25% of their annual income through sale of cowpea fodder (Dugje et al., 2009). As a proportion of income, the selling price of cowpea fodder may vary between 50 and 80% of the grain price (Singh et al., 2003). Importantly, changes in crop residue to primary product value alert crop improvement programs about changing demands and trait preferences (Blümmel and Rao, 2006; Sharma et al., 2010; Teufel et al., 2010). Price differentiations within residues of specific crops, such as in sorghum stovers, wheat, and rice straws, traded at the same time and place, as observed by Blümmel and Rao (2006) and Teufel et al. (2010), suggest that crop residue fodder quality rather than only harvest indices needs to be considered in crop improvement programs (Sharma et al., 2010; Erenstein et al., 2013).

In the current fodder market survey groundnut haulms were consistently priced higher than cowpea haulms and the value of the former relative to grain was 0.93 while that of the latter was 0.30. These findings are contrary to findings from fodder market surveys by Ayantunde et al. (2014) in Mali and Sapna et al. (2016a) in Niger, where cowpea haulms were always priced higher than groundnut haulms. However, in the current survey, the cowpea fodder quality traits of N, NDF, ADF, IVOMD, and ME where consistently inferior to those of groundnut haulms (**Table 2**), whereas the reverse was true in the Mali and Niger surveys (Ayantunde et al., 2014; Sapna et al., 2016a). Further Sapna et al. (2016a) reported that feed and fodder prices were significantly (*P* < 0.05) correlated with key laboratory fodder quality traits with nitrogen, ME IVOMD, and NDF. The findings from Nigeria, Mali, and Niger are therefore consistent in suggesting that haulm fodder quality is the guiding principle for pricing. Accepting these premises, the following proposition can be made for cowpea improvement: the observed cowpea haulm value of roughly 30% that of the cowpea grain could be increased if cowpea haulm quality could be increased to equal that of groundnut haulms.

### Exploitable Variations in Grain and Haulm Yield

Very substantial variations were observed among the 50 genotypes in grain and haulm yields, which varied about threefold (**Table 3**). Observed grain yields, except for those at Magaria, generally agreed with other reports from Northern Nigeria where yields averaged 737 kg/ha with one application of insecticide at flowering time and 904 kg/ha with two applications (Ajeigbe and Singh, 2006). These studies report higher yields than the reported average grain yield of 465 kg/ha for West Africa (FAO, 2008) and the average of 265 kg/ha for farmers’ field in Niger (JAICAF, 2009). Haulm yields in the present study were greater than the mean yields for nine cowpea varieties tested at Niamey, Niger (Atta et al., 2011). However, grain and haulm yields in all the locations in the current study were lower than the average yield of 1.3 tons and 2.5 tons/ha recorded for average grain and haulm yield, respectively, in farmers’ field using IT89KD-288 a promising dual purpose variety in Nigeria (Singh et al., 2003). Yields of grains and haulms reaching close to 7 tons/ha each have been reported from Ethiopia (Ayana et al., 2013). It is important to point out that several sites in the current study, such as Magaria and Malamadori are within the Sahelian agro-ecology, which has characteristically low rainfall, poor soil fertility, and striga attack. These factors depress grain and haulm yields. The diversity in agro-ecological zones also explains the G × E effects observed (**Table 4**). Overall pooled grain to haulm ratio was around 2.2, while the variation across the locations was wider (1.2–11.9) than across years (2.1–2.5). Narrower grain to haulm ratios of 0.73–1.00 have been reported for top ten accessions of cowpea bred specifically for high grain yields in Ethiopia where the grain yields were almost 4.6–6.5 times the yields obtained in the present study (Ayana et al., 2013). Similarly, significant differences in grain yield among the 60 cowpea genotypes were reported by Dalsaniya et al. (2009).

### Exploitable Variations in Haulm Fodder Quality Traits in Cowpea

Significant G × E effects was also observed for cowpea haulm fodder quality traits (see **Tables 3** and **4**) even though genotypic variations in fodder quality were always highly significant. Similar findings of significant effects of genotype and genotype by location interactions on haulm yield were reported for nine varieties tested over two locations in Niamey by Atta et al. (2011). For differences in laboratory fodder quality traits to matter, they need to be nutritionally significant for livestock (Sharma et al., 2010). Laboratory fodder quality traits are proxies since true fodder quality is ultimately only reflected in meat, milk and fiber production and draught power output. Laboratory analyses are indispensable short cuts because animal experimentation is unsuitable for routine feed quality investigations, particularly where many entries are involved as is the case for crop improvement (Sharma et al., 2010). Laboratory fodder quality traits used in the present work relate to key nutrient requirements of livestock such as protein and energy, while the cell wall constituents NDF, ADF, and ADL all contribute negatively to overall IVOMD and ME (Van Soest, 1994). In contrast to cereal crop residues, which generally fall short of the minimum N content of 1–1.2% needed for ruminant microbes to efficiently digest feed in the rumen, legume haulms contain much more than the minimum N and can therefore serve as supplements to cereal crop residues. Nitrogen content of the haulms in the present study ranged from 1.8 to 3.0%, similar to reported values of 2.3 by FAO (1981), 1.3–3.5 by Kaasschieter et al. (1998), and 2.4–3.5 by Anele et al. (2011). Where legume haulms are mainly used as supplements, exploitable variations in N content would be of primary interest. Cowpea haulm N content varied with genotype by 1.7-fold (**Table 3**), or by one percentage point, itself equivalent to the minimum microbial N requirement. Thus, if cowpea haulms were used to supplement cereal stover containing 0.60% N to reach a dietary content of 1.1% N, the diet would need to contain only 26% cowpea haulms with a N content of 2.5%, in contrast to a diet of 56% haulms if they contained only 1.5% N. Such differences have huge implication in practical feeding and economics. While, haulm N tended to be inversely associated (*r* = -0.26, *P* = 0.07) with grain yield (**Table 5**), an association also observed in groundnut (Blümmel et al., 2012b), the relationship was weak and haulm N content of about 2.4% can be realized without seriously jeopardizing grain yield.

Significant variation in average IVOMD of cowpea haulms across genotypes, locations and years were observed (**Table 3**). An *ex ante* assessment by Kristjanson and Zerbini (1999) postulated that a one percent increase in digestibility of sorghum/pearl millet stover would lead to 6–8% greater outputs in milk, meat or draught power. This *ex ante* assessment was broadly supported by fodder market studies of sorghum stover reporting that a one percent unit increase in IVOMD was associated with a stover price premium of about 5% (Blümmel and Rao, 2006). Anandan et al. (2010) showed that feeding total mixed rations differing only in the quality of the basal sorghum stover (IVOMD of 47 and 52%, respectively) resulted in differential milk potential of 5 kg per day in dairy buffalo. In other words, differences in IVOMD of 3–5% units are important to livestock productivity.

### Approaches to Multi-Trait Selection in Cowpea

**FIGURE 1:**
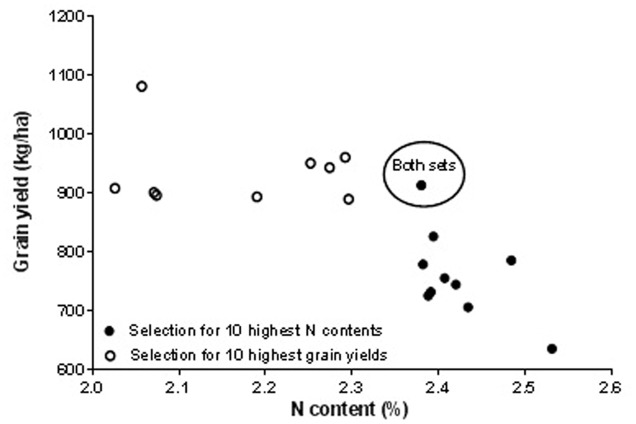
**Effect of first choice in multi-trait selection: grain yield versus haulm nitrogen (N) content**.

**FIGURE 2:**
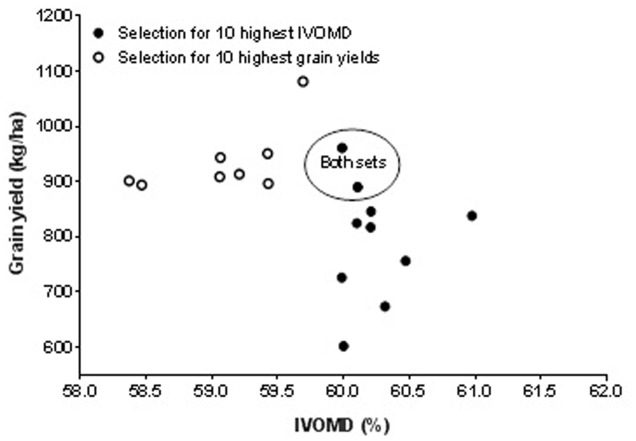
**Effect of first choice in multi-trait selection: grain yield versus *in vitro* organic matter digestibility (IVOMD)**.

**FIGURE 3:**
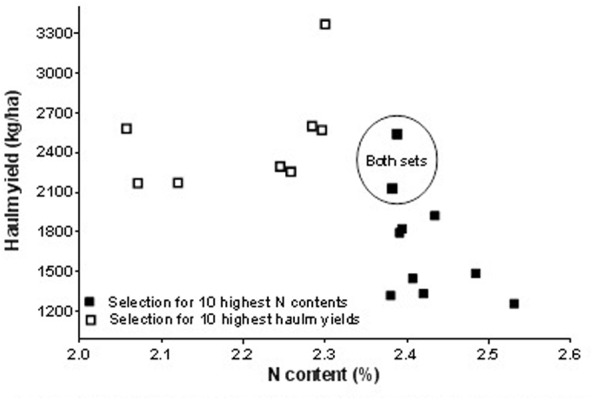
**Effect of first choice in multi-trait selection: haulm yield versus haulm nitrogen (N) content**.

**FIGURE 4:**
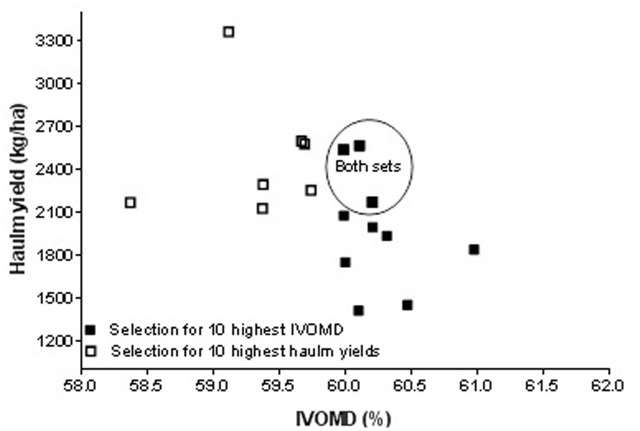
**Effect of first choice in multi-trait selection: haulm yield versus haulm IVOMD**.

Approaches to multi-trait selection in cowpea are presented in **Figures 1**–**4** that depict selections for either high grain or haulm yield with selection for high N and IVOMD. Overlap always occurred among the 10 highest performers in each category. In other words, selections were never exclusive. Among the 50 screened genotypes, selection of the top ten grain and haulms yielders both resulted in three genotypes- IT07K-291-69, IT06K-270, and IT98K- 412-13 with both high grain and haulm yields that could potentially to be recommended as dual-purpose genotypes for adoption by farmers. While the very highest grain and haulm yielders did not have the highest haulm N or IVOMD, they were better than the average in haulm fodder quality. The grain: haulm price ratio in cowpea reported in **Table 1** suggests that grain yield should be the primary trait in cowpea improvement. Grain and haulm yields only tended (*P* = 0.07) to be positively associated (*r* = 0.26), which agrees with the association observed by Grings et al. (2012) of *r* = 0.27. Such relationship is too weak to automatically increase haulm yield with selection for grain yield, and therefore haulm yield needs to be targeted as additional trait. In fact, it is conceivable that haulm yield might become the primary trait with an altered grain: haulm price ratio. To illustrate the likely impact of this effect, income from the top three lines with either highest grain and/or highest haulm yields were used to calculate income from sale of grains and haulms at constant grain prices of 97 Naira (0.64 USD) per kg across Nigeria, Niger, and Mali. The price ratio of grain to haulm of 3.5:1 obtained in the market survey in Northern Nigeria was used for Nigeria while the price ratio of 2.4:1 reported by Sapna et al. (2016b) was used for Niger and the 0.6:1 reported by Ayantunde et al. (2014) was used for Mali. Results of the calculations show that a mere change in price ratio of grain to straw for the highest grain yielders improved the total income by 1.1 and 2.8 fold in Niger and Mali, respectively and for the highest haulm yielders the total income increased by 1.2 and 2.4 in Niger and Mali, respectively (**Table 6**). Apart from the changes in the income, changes in price ratios of grain to straw resulted in changes in income from haulms to grains. In general, the proportional income from haulms increased with a lower price ratio of grain to straw (**Table 6**). Thus in Niger and Mali income from cowpea cropping could be greater from cultivars with haulm yield as the primary and grain yield as the secondary trait.

**Table 6:** Implications of changes in price ratio of grain to straw on potential income from sale of grains and haulms at constant grain prices across Nigeria, Niger, and Mali.

	Yields kg/ha		Crop value US $								
			Nigeria			Niger			Mali		
High grain lines	Grains	Haulms	G($)	H($)	Total ($)	G($)	H($)	Total ($)	G($)	H($)	Total ($)
IT07K-291-69	1081	2580	688	498	1186	687	687	1374	690	2760	3450
IT98K-1263	961	2080	618	395	1013	617	547	1164	625	2215	2840
IT03K-316-1	951	1640	609	314	923	604	438	1042	614	1748	2362
Average	998	2100	645	395	1040	644	549	1193	663	2221	2884

Overall across locations income proportions grain: straw is 38:62.											

	**Yields kg/ha**		**Nigeria**			**Niger**			**Mali**		
**High haulm lines**	**Grains**	**Haulms**	**G($)**	**H($)**	**Total ($)**	**G($)**	**H($)**	**Total ($)**	**G($)**	**H($)**	**Total ($)**

IT99K-377-1	803	3370	510	649	1159	505	898	1403	494	3623	4117
IT99K-573-1-1	701	2600	445	502	947	454	681	1135	453	2777	3230
IT07K-291-69	1081	2580	688	498	1186	687	687	1374	690	2760	3450
Average	862	2850	549	549	1098	548	756	1304	540	2059	2599
Overall across locations income proportions grain: straw is 27:73.											

There are more opportunities for whole plant value optimization through multi-trait cowpea improvement. It is highly likely that the reason for the higher prices of groundnut compared to cowpea haulms observed in Nigerian fodder markets (**Table 1**) resides in the higher haulm fodder quality of groundnut haulms (**Table 2**) in these markets. The fact that the reverse was true for fodder markets in Niger (Sapna et al., 2016a) and Mali (Ayantunde et al., 2014), that is cowpea haulms were more expensive than groundnut haulms with cowpea haulms having superior fodder quality, is suggestive of “fodder quality” playing a decisive role in pricing. In the present work, the average N content of traded cowpea and groundnut haulms was 2.0 and 2.4%, respectively (**Table 2**). The findings in **Figures 1** and **3** shows that cowpea lines exist that have 2.4% nitrogen in the haulms without serious trade-offs in grain or haulm yields. The same holds true for haulm IVOMD where cowpea line exists that surpass those found in traded groundnut haulms without serious trade-offs with grain or haulm yields (see **Figures 2** and **4** and compare with **Table 2**). If such cowpea haulms could be sold at groundnut haulm prices, the overall value of the cowpea plant would substantially increase with a greater proportion of income coming from haulm sales.

## Conclusion

Given the fact that cowpea is a major legume crop cultivated in West Africa with an ability to meet food, feed and fertilizer needs through grain, fodder and nitrogen enrichment, greater emphasis is required for the development of full purpose cowpea genotypes. Absence of a negative correlation between grain and fodder yields; positive correlations between grain/fodder yield and fodder quality attributes coupled with wide variations in yield and fodder quality attributes in tested cowpea lines provides the ideal conditions for developing full purpose cowpea genotypes. Demand domains for cowpea in terms of yields of grains, haulms, quality of haulms and potential economic consequences are quite variable across the West Africa region. Understanding the demand domains and matching the cowpea lines specific to a particular domain is key to improving the economics of cowpea cropping. The availability of full purpose cowpea genotypes with multi-traits vis-à-vis conventional breeding approaches to promote superior grain or haulm lines offers wider scope over a larger domain and user base. The growing demand for livestock products along with feed shortage in the West African region calls for greater attention by cowpea breeders and livestock nutritionists to collaborate on exploiting the existing genetic variation in yields and fodder quality attributes of cowpea to improve incomes and livestock productivity. This will lead to better crop and livestock integration, which is so important in West Africa.

## Author Contributions

MB, IO, OB, CF, and AS designed the research. OB and CF were involved in the field trails and interpretation of agronomic data. EG and IO were involved in designing, conducting, and interpreting the market survey. PK was involved in the fodder quality analysis and its interpretation. RD was involved in the statistical analysis and its interpretation. MB and AS were involved in the writing of manuscript with contributions from all co-authors.

## Conflict of Interest Statement

The authors declare that the research was conducted in the absence of any commercial or financial relationships that could be construed as a potential conflict of interest. The handling Editor declared a shared affiliation, though no other collaboration, with one of the authors (OB) and states that the process nevertheless met the standards of a fair and objective review.
